# Multidisciplinary and multifaceted outpatient management of patients with osteoarthritis: protocol for a randomised, controlled trial

**DOI:** 10.1186/1471-2474-11-253

**Published:** 2010-11-01

**Authors:** Rikke Helene Moe, Till Uhlig, Ingvild Kjeken, Kåre Birger Hagen, Tore Kristian Kvien, Margreth Grotle

**Affiliations:** 1 National resource centre for rehabilitation in rheumatology, Dept. of Rheumatology, Diakonhjemmet Hospital, Oslo, P.O.Box 23 Vinderen, 0319 Oslo, Norway

## Abstract

**Background:**

Osteoarthritis (OA) is a prevalent joint disorder with a need for efficient and evidence-based management strategies.

**Objectives:**

The primary purpose of this study is to compare the effects of a multidisciplinary outpatient clinic, including a brief group-based educational programme, with a traditional individual outpatient clinic for patients with hip, knee, hand or generalized OA. A secondary purpose is to investigate the effects of a telephone follow-up call.

**Methods:**

This is a pragmatic randomised single-blind controlled study with a total of 400 patients with hip, knee, hand or generalized OA between 40 and 80 years referred to an outpatient rheumatology hospital clinic. The randomisation is stratified according to the diagnostic subgroups. The experimental group is exposed to a multidisciplinary and multifaceted intervention, including a 3.5 hour group-based patient education programme about OA in addition to individual consultations with members of a multidisciplinary team. The control intervention is based on regular care with an individual outpatient consultation with a rheumatologist (treatment as usual). Primary outcomes are patient satisfaction measured at 4 months and cost-effectiveness measured at 12 months. Secondary outcomes are pain and global disease activity measured on a numeric rating scales (NRS), generic and disease specific functioning and disability using Short Form-36 (SF-36) health survey, the Western Ontario and McMaster Universities Osteoarthritis Index 3 (WOMAC), the Australian/Canadian Osteoarthritis Hand Index (AUSCAN), and a patient-generated measure of disability (Patient-Specific Functional scale, PSFS). Global perceived effect of change in health status during the study period is also reported. At 4-month follow-up, patients in both groups will be randomly allocated to a 10-minute telephone call or no follow-up ("treatment as usual"). After additional 8 months (12-month follow-up) the four groups will be compared in a secondary analysis with regard to health outcomes and health care costs.

**Discussion:**

This trial will provide results on how multidisciplinary and multifaceted management of patients with OA affects health outcomes and health care costs.

**Trial registration:**

Current Controlled Trials ISRCTN25778426

## Background

Osteoarthritis (OA) is the most prevalent joint disorder and is associated with pain, functional disability and impaired quality of life [1]. The degenerative disease process of synovial joints leads to radiographic signs and the clinical symptoms like pain, stiffness and reduced joint motion. OA is strongly associated with aging, has most commonly an onset after the age of 50 years, and is ranked as the fourth most common condition in older women and the eight most common in older men [2-4].

OA poses significant burdens in terms of reduced general health [5], work disability [6], and large societal costs which may be comparable to rheumatoid arthritis and other inflammatory diseases [7-9]. With an increasing proportion of older people in the population, OA constitutes a growing public health problem. Effective and evidence-based preventive and treatment strategies for OA are important as they may reduce both the individual burden of OA, and the economic burden to the society.

Recommendations for management of OA focus on a combination of pharmacological and non-pharmacological treatments [3,10,11]. Most of the non-pharmacological treatments have been studied in patients with hip and knee OA with a special focus on exercise, physical activity, patient education and weight control [2]. Reduced pain and improved function has been documented in patients with knee OA, and exercises and information are considered as important non-pharmacological interventions for this patient group.

Non-pharmacological management of hand OA and generalized OA has been investigated in only a few studies [12]. One recent systematic review concerning treatment of hand OA included 31 studies, most of them on the effect of pharmacological treatment [12]. Even though a few of the treatment strategies did show effect in terms of reduced pain and improved function, methodological weaknesses made direct recommendations on treatments for hand OA impossible. Various patient education programmes have been used for treating chronic health conditions, but no consensus has been reached on how patient education should be applied; either using a disease specific or generic approach, group-based or individual education, and either given by health professionals or lay tutors [13,14].

Standard treatment for OA in specialist care is an individual consultation with a rheumatologist, sometimes followed by referral to one or several other health professionals. However, many patients with OA experience this provision of health services as fragmented. Due to an increasing demand for a more coordinated approach, a pilot multidisciplinary OA outpatient clinic was established at Diakonhjemmet Hospital, Oslo, Norway, in 2003. The multidisciplinary team included rheumatologists, nurses, health secretaries, occupational therapists, physical therapists, pharmacists, orthopaedic surgeons and a dietician. Over a two year period, approximately 300 patients with OA were referred to the OA clinic by their general practitioners or specialists. Of these 300, 73 patients were followed for 1 year [15]. Most (78%) of these patients were women (mean age 61 years (range 24-92)). More than 60% reported to have pain in their knees and/or hands, while 40% had pain primarily localized to the hip. Approximately one fourth of the patients used NSAIDS and/or other types of analgesics. At the one year follow-up, 95% of the patients were very satisfied with the team care received, and pain and functioning was moderately improved [15]. This first evaluation with an open longitudinal design without a control group warranted the need for a more systematically and thorough evaluation of the effect and cost-benefits of this multidisciplinary, coordinated outpatient OA clinic.

The number of referrals to the clinic increased in the pilot period. To meet patients' need for information, a brief group-based educational programme was developed. Thus, in the updated multidisciplinary OA clinic, starting from 2005, patients first receive a 3.5 hours group education. After a lunch brake, they have individual consultations with a rheumatologist and thereafter, dependent on identified needs, also have encounters with other health professionals.

Few other studies have evaluated the effect of group-based educational programmes for OA in combination with a pragmatic individual approach, especially in a setting of a randomised clinical trial (RCT) [13].

The purposes of this study are 1) to compare the effect of a multidisciplinary outpatient clinic, including a brief group-based educational programme, with a regular care individual outpatient clinic for patients with hip, knee, hand and/or generalized OA, and 2) to compare the effect of a telephone follow-up call with follow-up "as usual" for patients with hip, knee, hand and/or generalized OA.

### Hypotheses

1) Patients with hip, knee, hand and/or generalized OA who receive a multidisciplinary and multifaceted intervention will be more satisfied with the health service at 4-month follow up compared to patients who receive individual consultation(s) in a regular care individual outpatient clinic.

2) The multidisciplinary and multifaceted outpatient clinic will reduce the use of health services and thus, this intervention will be more cost-effective at 12-month follow up than the traditional individual outpatient clinic.

3) Patients who receive a telephone call after 4 months will at 12-month follow-up report more satisfaction with the health service than those within the same group allocation who do not receive any telephone follow-up.

4) At 4- and 12-month follow-up, changes in the secondary outcomes VAS pain, global disease activity, generic and disease specific functioning and disability (measured by SF-36, WOMAC and AUSCAN) will not be different between patients who are managed in a multidisciplinary outpatient clinic and patients who receive regular care in a traditional individual outpatient clinic.

## Methods

Patients are randomised into the two interventions within three strata: patients with primary hand, hip or knee problems. A project coordinator includes patients and coordinates the patient-reported data collection with questionnaires. Health professionals who are taking part in the two interventions are not involved in the data collection. Blinding of providers and patients is not possible in this type of study, but the outcome assessors as well as those who perform the follow up telephone call are blinded. All patients involved in the project have signed an informed consent and have been informed according to the Helsinki declaration. The data inspectorate and the regional ethical committee (REK) reviewed and approved the project (REK ref 156-06073 1.2006.598); the ISRCTN trial number is 25778426.

### Study sample and setting

A total of 400 participants referred to an outpatient hospital rheumatology clinic with a diagnosis of hip, knee, hand and/or generalized OA, will be recruited for the study.

### Subjects

To be eligible for inclusion, participants must be between 40 and 80 years and have a clinical diagnosis of hip, knee, hand and/or generalized OA confirmed by a specialist. The eligibility criteria are described in Table 1.

**Table 1:** Eligibility criteria

Inclusion criteria	Exclusion criteria
Clinical diagnosis osteoarthritis	Recent surgery
Referral from general practitioner or specialist	Cognitive impairments
40-80 years	Inability to read and understand Norwegian
	Recent trauma in the extremities, other known or relevant co-morbidities such as rheumatoid arthritis and cancer

### Primary intervention

The *experimental intervention *is a multidisciplinary and multifaceted intervention, in which the referred patients first receive a 3.5 hour group-based educational programme, and thereafter individual consultations with a rheumatologist and other members of the multidisciplinary team, dependent on identified needs. The multidisciplinary team consists of rheumatologists, an orthopaedic surgeon, nurses, health secretaries, physiotherapists, occupational therapists, a pharmacist and a dietician.

The group-based educational programme contains four main themes identified through qualitative interviews with patient advocates. The themes are: a) What is OA? b) Activity possibilities or limitations, what can we do ourselves? c) Treatment options and d) How to live with OA.

The *control intervention *is regular care in an individual outpatient clinic. The patients are referred to and examined by a rheumatologist. According to usual procedures they are referred to other specialists or health professionals such as orthopaedic surgeons, physiotherapists, occupational therapists etc. dependent on identified needs. The access to health care providers is in principle similar to the access in the experimental group, but encounters occur according to referral.

### Secondary intervention

Patients in both the primary groups are randomised to either a short 'telephone call' or 'no telephone call' after they have completed the 4-month follow-up assessment. Patients who receive 'no telephone call' are treated as usual, which imply that they can contact the outpatient clinic if they have additional questions or comments. Patients who receive the 'telephone call' are asked the following three questions: "how are you doing?", "did you receive the help you needed for your OA when you were at the hospital?", and "do you want to add any further information concerning your health status and the treatment given"?

### Recruitment procedure

All referrals from general practitioners to the outpatient clinic with focus on OA are screened for eligibility according to the inclusion and exclusion criteria. A research coordinator contacts patients who are potentially eligible for the study by telephone, inform them about the study and ask whether they want to participate. In the same telephone contact, a screening tool for classification of OA is used in the cases in which an OA diagnosis was evident based on information in the referral letter [16]. If the OA diagnosis cannot be clinically confirmed during the examination by the rheumatologist during the first appointment, the participant will be excluded. Written consent according to the declarations of Helsinki is obtained before enrolment to the study. In addition, they are sent the first questionnaire by mail to be filled in before the first appointment at the outpatient clinic.

### Group allocation

All patients are randomly allocated to one of two groups, experimental or control group. This randomisation is performed after completion of the baseline questionnaires. To ensure equal-sized treatment groups, random permuted blocks of 4-8 participants are used [17]. The stratified random allocation schedule is computer generated by a statistician who is not otherwise involved in recruitment, assessment or treatment of participants. The randomisation sequence is carried out in sequentially numbered, opaque, sealed envelopes.

### Follow-up procedures

The participants are at the 4-month follow-up asked to complete a mailed questionnaire, and return it by mail. After completion of the questionnaire, participants are randomly allocated to either a brief 'telephone call' of approximately 10 minutes or follow-up "as usual", which means that the patients may contact the clinic if they need to.

The final follow-up is a mailed questionnaire at 12 months, which is mailed back to the project group after completion. In addition to these two mailed follow-up questionnaires, the participants complete a monthly diary regarding use of health care.

**Figure 1:**
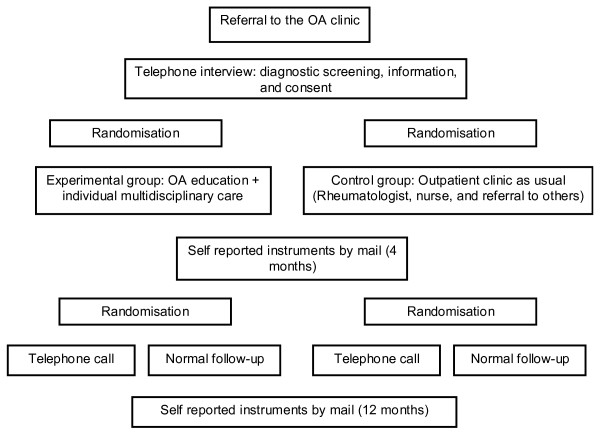
**Design and assessments in the study**.

The administration of questionnaires is carried out by a person not otherwise involved in the treatment of participants (research coordinator AF). The telephone calls are carried out by two researchers (IK, ES) who are blinded to group allocation. Figure 1 provides an overview of the design and follow-up assessments.

## Outcome measures

Table 2 provides an overview of the various questionnaires used at data collection at baseline, after 4 and 12 months.

**Table 2:** Questionnaires and assessments in the study

	At inclusion	4 mnd	12 mnd
Socio-demographic data			
Age, gender, height, weight, marital status	X		
Work situation (profession, % of full time)	X		
Education	X		
Smoking	X		
Physical activity	X		
Earlier and current treatments	X		
Co-morbidities	X		
Pain and functioning			
Pain during the last week (NRS 0-10)	X	X	X
WOMAC/AUSCAN	X	X	X
PSFS	X	X	X
Self-efficacy/ASES	X	X	X
Health related quality of life			
SF-36	X	X	X
EuroQoL	X	X	X
Satisfaction with care			
Satisfaction with care (numeric rating scale 0-10)		X	X
Various data at follow-up			
Use of health care and services (diary)		X	X
Global question on change of health status		X	X

NRS = Numeric Rating Scale

SF-36 = Short Form-36 health survey

WOMAC = Western Ontario and McMaster Universities Osteoarthritis Index 3 AUSCAN = Australian/Canadian Osteoarthritis Hand Index

PSFS = Patient-Specific Functional scale

ASES = Arthritis Self Efficacy Scale

### Primary outcomes

The primary outcome measure is patient satisfaction with the health service, which will be evaluated on a 11 point numeric rating scale (NRS) from 0 = not satisfied to 10 = very satisfied [18]. The co-primary outcome is c*ost-effectiveness*, which will be based on changes in utility-scores based on EQ-5D in the denominator [19] and direct and indirect costs related to OA in the numerator. The costs will be assessed by recording the use of health care by patient self-report. All patients will receive a diary and all consultations and costs related to OA care during the follow-up will be recorded in this diary. Indirect costs related to the disease, like sick leave, lost working days and the use of social security will also be included in this cost-effectiveness analysis.

### Secondary outcomes

Secondary outcomes are pain and global disease activity, generic and disease specific functioning and disability using the component summary scales in the SF-36 health survey, WOMAC, AUSCAN, a patient-generated measure of disability (PSFS), and global perceived effect of change in health status during the study period.

*Pain *is measured on a NRS (range 0-10) with the anchor words 'no pain at all' and 'unbearable pain'. *Global disease activity *is measured on a NRS (0-10) with the anchor words 'no symptoms' and 'extremely severe', answering the question: "All symptoms taken into account, what do you think about your condition the last week?"

Health related quality of life is measured by the generic questionnaire SF-36 [20]. The SF-36 Health Survey is a widely used generic instrument that comprises eight health scales that contribute to two higher order health scales, the Physical Component Summary (PCS) and Mental Component Summary (MCS) summary scores which give a mean (SD) of 50 based on normative data from the general population (Ware, 1995). The SF-36 is often used to assess health related quality of life in the general population and in different diseases. The English version has been translated to and validated in Norwegian [21].

Disease specific functioning in daily life is assessed by the WOMAC [22] for patients with hip or knee OA. This instrument is responsive and valid for measuring pain (5 items), stiffness (2 items) and physical function (17 items). The Likert scale version of the scale is used with response options none, mild, moderate, severe, extreme.

For patients with hand OA disease specific health problems are self-reported by the AUSCAN, which comprises 15 items relating to hand stiffness (one item), pain (five items) and problems with performance of activities (nine items). This study used the five-point descriptive scale version of the AUSCAN with response options none, mild, moderate, severe, extreme [23].

Through the use of the *PSFS*, all patients will also be asked to describe the three most important activity limitations caused by their OA and score each of these on a 0-10 point scale according to the ability to perform these activities [24]. Patients will be interviewed regarding their activity limitations during the telephone interview at baseline before randomisation.

The *global perceived effect of change *in health status during the study period will be assessed on a 5-point scale ranging from much better through unchanged to much worse.

*Self-efficacy *will be evaluated using the Arthritis Self Efficacy Scale (ASES) for pain and functioning [25].

### Baseline questionnaire

All patients are asked to fill in a questionnaire at inclusion, containing information on socio-demographics (age, gender, height, weight, marital status, work situation, emotional distress and physical activity), earlier and current pharmacological and non-pharmacological treatment for their OA and co-morbidities. They are also asked to complete standardised instruments for measurement of pain; functioning and health related quality of life.

Additional therapies and co-interventions are also registered. Patients who withdraw from the study will be registered by a research secretary. Delivery of interventions in both groups will be registered by the health professionals responsible for the intervention. Registering co-interventions will be part of the diary.

### Data analysis

#### Power calculations

As no satisfactory data on patient satisfaction and cost-effectiveness were available, power calculations were based on the secondary outcome pain with data from the pilot study and previous research on OA from our group [26-28]. In this RCT we compare two independent equally sized groups. We assume comparable variation in both groups, set alpha to 5%, beta to 80%, and assume a smallest clinical important difference between the groups of 0.20 based on an estimated difference for pain measured by WOMAC/AUSCAN. The standardized difference (0.20)/SD (0.70)) will be 0.25. According to the Altman nomogram, approximately 200 patients will be needed in each of the two treatment groups.

#### Statistical analyses

Primary data analysis will be performed in a blinded manner, by conducting the effect analyses without knowing which intervention each of the two groups received. The treatment groups will be examined for baseline comparability. Both parametric and nonparametric statistical analyses will be used, dependent on how the variance is distributed. Effect of the interventions will be analyzed according to intention-to treat principles. Further, exploratory analyses will be performed to study the treatment effect within the three subgroups hip, knee and hand OA.

## Discussion

To our knowledge, this will be one of the first RCT's that will examine how a multidisciplinary approach affects treatment outcomes for patients with OA. Primary outcome will be patient satisfaction with health service assessed at 4 months, while, cost-effectiveness will be the co-primary outcome assessed at 12 months, and various aspects of health status are the secondary outcomes at both time-points.

A rheumatologist and a project coordinator examine all referrals to the multidisciplinary OA clinic at Diakonhjemmet Hospital. Patients aged 18-80 years are recruited to the study by a screening-form for OA based on clinical criteria of OA in hand hip or knee. Patients who may be eligible for the study receive oral and written information about the methods and aims of the study and are informed that participation is voluntary and can be terminated at any time without stating a reason and without any impact on their care. Informed consent will be obtained from all patients. They receive questionnaires by mail before the clinical examinations and if they have problems answering questions, a project assistant at the first encounter provide assistance. A smaller self-reported questionnaire on function, pain and health status will be mailed to the patients for the 4- and 12-month assessments.

All health professionals in the multidisciplinary team are represented among the staff at the Department of Rheumatology and a research secretary will be responsible for performance of practical aspects and organisation.

### Time schedule

Data collection is ongoing, and will be completed within 2011 which will be followed by a one-year period of data analyses and manuscript preparation.

The project will, by means of a randomised controlled trial, contribute to new knowledge on the effect of patient education for patients with OA. The project will also contribute with cost-effectiveness analyses comparing the two different methods of outpatient care.

## Competing interests

The authors declare that they have no competing interests.

## Authors' contributions

MG, TU, KBH, and TKK developed the study design. All authors have taken active part in planning the performance of the study, developing the intervention, the analytic approach and writing the manuscript for the protocol. All authors have read and approved the final manuscript.

## Pre-publication history

The pre-publication history for this paper can be accessed here:

http://www.biomedcentral.com/1471-2474/11/253/prepub

## References

[B1] Arden N Nevitt MC Osteoarthritis: epidemiology Best Pract Res Clin Rheumatol 2006 20 1 3 25 10.1016/j.berh.2005.09.007 16483904

[B2] Jordan KM Arden NK Doherty M Bannwarth B Bijlsma JW Dieppe P Gunther K Hauselmann H Herrero-Beaumont G Kaklamanis P Lohmander S Leeb B Lequesne M Mazieres B Martin-Mola E Pavelka K Pendleton A Punzi L Serni U Swoboda B Verbruggen G Zimmerman-Gorska I Dougados M EULAR Recommendations 2003: an evidence based approach to the management of knee osteoarthritis: Report of a Task Force of the Standing Committee for International Clinical Studies Including Therapeutic Trials (ESCISIT) Ann Rheum Dis 2003 62 12 1145 55 10.1136/ard.2003.011742 14644851 PMC1754382

[B3] Zhang W Doherty M Arden N Bannwarth B Bijlsma J Gunther KP Hauselmann HJ Herrero-Beaumont G Jordan K Kaklamanis P Leeb B Lequesne M Lohmander S Mazieres B Martin-Mola E Pavelka K Pendleton A Punzi L Swoboda B Varatojo R Verbruggen G Zimmermann-Gorska I Dougados M EULAR evidence based recommendations for the management of hip osteoarthritis: report of a task force of the EULAR Standing Committee for International Clinical Studies Including Therapeutics (ESCISIT) Ann Rheum Dis 2005 64 5 669 81 10.1136/ard.2004.028886 15471891 PMC1755499

[B4] Heinegard D Johnell O Lidgren L Nilsson O Rydevik B Wollheim F Akesson K The Bone and Joint Decade 2000-2010 Acta Orthop Scand 1998 69 3 219 20 10.3109/17453679809000917 9703390

[B5] Hawley DJ Wolfe F Pain, disability, and pain/disability relationships in seven rheumatic disorders: a study of 1,522 patients J Rheumatol 1991 18 10 1552 7 1837315

[B6] Pincus T Mitchell JM Burkhauser RV Substantial work disability and earnings losses in individuals less than age 65 with osteoarthritis: comparisons with rheumatoid arthritis J Clin Epidemiol 1989 42 5 449 57 10.1016/0895-4356(89)90135-2 2732773

[B7] Rothfuss J Mau W Zeidler H Brenner MH Socioeconomic evaluation of rheumatoid arthritis and osteoarthritis: a literature review Semin Arthritis Rheum 1997 26 5 771 9 10.1016/S0049-0172(97)80044-3 9144852

[B8] Maetzel A Ferraz MB Bombardier C A review of cost-effectiveness analyses in rheumatology and related disciplines Curr Opin Rheumatol 1998 10 2 136 40 10.1097/00002281-199803000-00009 9567209

[B9] Gabriel SE Crowson CS Campion ME O'Fallon WM Direct medical costs unique to people with arthritis J Rheumatol 1997 24 4 719 25 9101508

[B10] Zhang W Doherty M Leeb BF Alekseeva L Arden NK Bijlsma JW Dincer F Dziedzic K Hauselmann HJ Herrero-Beaumont G Kaklamanis P Lohmander S Maheu E Martin-Mola E Pavelka K Punzi L Reiter S Sautner J Smolen J Verbruggen G Zimmermann-Gorska I EULAR evidence based recommendations for the management of hand osteoarthritis: report of a Task Force of the EULAR Standing Committee for International Clinical Studies Including Therapeutics (ESCISIT) Ann Rheum Dis 2007 66 3 377 88 10.1136/ard.2006.062091 17046965 PMC1856004

[B11] Zhang W Moskowitz RW Nuki G Abramson S Altman RD Arden N Bierma-Zeinstra S Brandt KD Croft P Doherty M Dougados M Hochberg M Hunter DJ Kwoh K Lohmander LS Tugwell P OARSI recommendations for the management of hip and knee osteoarthritis, part I: critical appraisal of existing treatment guidelines and systematic review of current research evidence Osteoarthritis Cartilage 2007 15 9 981 1000 10.1016/j.joca.2007.06.014 17719803

[B12] Towheed TE Systematic review of therapies for osteoarthritis of the hand Osteoarthritis Cartilage 2005 13 6 455 62 10.1016/j.joca.2005.02.009 15922179

[B13] Warsi A Wang PS LaValley MP Avorn J Solomon DH Self-management education programs in chronic disease: a systematic review and methodological critique of the literature Arch Intern Med 2004 164 15 1641 9 10.1001/archinte.164.15.1641 15302634

[B14] Mulligan K Newman S Psychoeducational interventions in rheumatic diseases: a review of papers published from September 2001 to August 2002 Curr Opin Rheumatol 2003 15 2 156 9 10.1097/00002281-200303000-00013 12598805

[B15] Moe RH Uhlig T Christensen BS Kvien TK A multidisciplinary osteoarthritis clinic: one year followup Ann Rheum Dis 2006 65 Suppl II 655 2006

[B16] Roux HC Saraux A Mazieres B Pouchot J Morvan J Fautrel B Testa J Fardellone P Rat CA Coste J Guillemin F Euller-Ziegler L Screening for hip and knee osteoarthritis in the general population: predictive value of a questionnaire and prevalence estimates Ann Rheum Dis 2008 67 10 1406 1411 10.1136/ard.2007.075952 18077540

[B17] Pocock SJ Clinical trials. A practical approach 1984 1 Chichester, John Wiley&Sons

[B18] Hewlett S Carr M Ryan S Kirwan J Richards P Carr A Hughes R Outcomes generated by patients with rheumatoid arthritis: how important are they? Musculoskeletal Care 2005 3 3 131 42 10.1002/msc.3 17042002

[B19] The EuroQol Group EuroQol--a new facility for the measurement of health-related quality of life Health Policy 1990 16 3 199 208 10.1016/0168-8510(90)90421-9 10109801

[B20] Ware JE Jr Sherbourne CD The MOS 36-item short-form health survey (SF-36). I. Conceptual framework and item selection Med Care 1992 30 6 473 83 10.1097/00005650-199206000-00002 1593914

[B21] Hagen KB Smedstad LM Uhlig T Kvien TK The responsiveness of health status measures in patients with rheumatoid arthritis: comparison of disease-specific and generic instruments J Rheumatol 1999 26 7 1474 80 10405932

[B22] Bellamy N Buchanan WW Goldsmith CH Campbell J Stitt LW Validation study of WOMAC: a health status instrument for measuring clinically important patient relevant outcomes to antirheumatic drug therapy in patients with osteoarthritis of the hip or knee J Rheumatol 1988 15 12 1833 40 3068365

[B23] Bellamy N Campbell J Haraoui B Buchbinder R Hobby K Roth JH MacDermid JC Dimensionality and clinical importance of pain and disability in hand osteoarthritis: Development of the Australian/Canadian (AUSCAN) Osteoarthritis Hand Index Osteoarthritis Cartilage 2002 10 11 855 62 10.1053/joca.2002.0837 12435330

[B24] Chatman AB Hyams SP Neel JM Binkley JM Stratford PW Schomberg A Stabler M The Patient-Specific Functional Scale: measurement properties in patients with knee dysfunction Phys Ther 1997 77 8 820 9 9256870 10.1093/ptj/77.8.820

[B25] Lorig K Chastain RL Ung E Shoor S Holman HR Development and evaluation of a scale to measure perceived self-efficacy in people with arthritis Arthritis Rheum 1989 32 1 37 44 10.1002/anr.1780320107 2912463

[B26] Kjeken I Dagfinrud H Slatkowsky-Christensen B Mowinckel P Uhlig T Kvien TK Finset A Activity limitations and participation restrictions in women with hand osteoarthritis: patients' descriptions and associations between dimensions of functioning Ann Rheum Dis 2005 64 11 1633 8 10.1136/ard.2004.034900 15829571 PMC1755278

[B27] Slatkowsky-Christensen B Kvien TK Bellamy N Performance of the Norwegian version of AUSCAN--a disease-specific measure of hand osteoarthritis Osteoarthritis Cartilage 2005 13 7 561 7 10.1016/j.joca.2005.02.013 15896986

[B28] Kjeken I Slatkowsky-Christensen B Kvien TK Uhlig T Norwegian version of the Canadian Occupational Performance Measure in patients with hand osteoarthritis: validity, responsiveness, and feasibility Arthritis Rheum 2004 51 5 709 15 10.1002/art.20522 15478169

